# The challenges of sequestering terrestrial carbon

**DOI:** 10.1093/nsr/nwac085

**Published:** 2022-05-19

**Authors:** Josep Peñuelas

**Affiliations:** Global Ecology Unit CREAF-CSIC-UAB, Spanish National Research Council (CSIC), Spain; Center for Ecological Research and Forestry Applications (CREAF), Spain

Nature may become a powerful ally in tackling climate change. Awareness is growing that ‘nature-based climatic solutions’ (NbCS) can help us to transition toward a society with zero net emissions and thus protect us from climate change and its consequent impacts while supporting biodiversity and securing ecosystem services. The actual potential of NbCS to provide the intended benefits, however, has not been rigorously assessed.

In an interesting, useful and timely exercise, Huang *et al.* have shed new light on this potential [1]. They have synthesized the available findings and used empirical models to consider target-oriented management (TOM), below-ground biomass and CO_2_ fertilization to assess China's sequestration of terrestrial carbon over 2010–2060 and its contribution to offsetting national CO_2_ emissions associated with energy. Huang *et al.* have concluded that China's terrestrial ecosystems could offset 12.2–15.0% and 13.4–17.8% of such peak CO_2_ emissions by 2030 and 2060, respectively. These results indicate that the role of sequestrating terrestrial carbon is substantial and cannot be underestimated for reaching carbon neutrality, but these percentages would be only about half what NbCS could provide, a quarter or more of the cost-effective mitigation needed by 2030 [2].

These percentages, however, remain uncertain due to the lack of complete information on how TOMs, below-ground biomass and the actual role of long-term CO_2_ fertilization in sequestering terrestrial carbon are driving simplifications in all these analyses. For example, these analyses assume simple linear increases in the coming decades in forest area, the area where grazing is excluded and the proportion of crop residue retained; use estimates of the amount of C sequestered in below-ground biomass using the mean root:shoot ratio when the actual ratio depends on forest type and climate; and account for the effect of CO_2_ fertilization without taking into account the likely limitations of nutrients, temperature and/or water with time or the effects of extreme events and the likely acclimation [3,4].

Several issues preclude a more optimistic expectation on enhanced sequestration of terrestrial carbon or at least pose substantial challenges in better developing these NbCSs. Huge areas of land would be needed for an effective long-term carbon sink to mitigate anthropogenic carbon emissions and the potential amount of carbon that these land areas could annually assimilate by photosynthesis is set by the amount of absorbed sunlight and available water, so large afforestation efforts may be unsustainable in arid and semi-arid regions such as Inner Mongolia [5]. The effectiveness of using these areas as a long-term carbon sink will depend on their ability to sustain a permanent carbon sink, the availability of adequate water and the competing needs for other ecological services, including the provision of food and fibre for the human population. Furthermore, converting land to forests or wetlands may have unintended costs, such as biophysical feedbacks that affect the local climate; the growth of plants that emit volatile organic carbon compounds, which are precursors to air pollution; or the production of prodigious amounts of methane by anaerobic wetlands.

The estimates of the future sequestration of terrestrial C in China by Huang *et al.* are very useful and a timely step forward in our understanding of NbCS, despite the uncertainties and the great challenges remaining. These estimates can orient not only the national policy of China but also the global policy, and warrant further research and steps for providing the information needed for a correct account of the evolution of the sequestration of terrestrial carbon in space and time and its role in offsetting CO_2_ emissions in the coming years. Facilitating these NbCS could clearly offer many additional benefits for people and biodiversity.

These results by Huang *et al.*, however, again indicate that the urgency for reducing the release of CO_2_ into the atmosphere and reaching carbon neutrality; the relatively low potential of converting solar energy to stored carbon; the vast amount of land needed to be effective carbon sinks; the factors conspiring against carbon sinks, especially water availability; and the risk of unintended consequences will require that efforts and resources be mostly aimed toward exploring additional possibilities of capturing carbon, but especially toward the clearest and most straightforward measure to reach carbon neutrality, i.e. reducing and eliminating carbon emissions associated with the combustion of fossil fuel, especially coal in China.
